# Identification of the oncogenic kinase TOPK/PBK as a master mitotic regulator of C2H2 zinc finger proteins

**DOI:** 10.18632/oncotarget.2735

**Published:** 2015-01-13

**Authors:** Raed Rizkallah, Paratchata Batsomboon, Gregory B. Dudley, Myra M. Hurt

**Affiliations:** ^1^ Department of Biomedical Sciences, Florida State University, Tallahassee, Florida, 32306, United States of America; ^2^ Department of Chemistry and Biochemistry, Florida State University, Tallahassee, Florida, 32306, United States of America

**Keywords:** Kinase, Mitosis, Phosphorylation, Zinc Finger Proteins, TOPK/PBK

## Abstract

TOPK/PBK is an oncogenic kinase upregulated in most human cancers and its high expression correlates with poor prognosis. TOPK is known to be activated by Cdk1 and needed for mitotic cell division; however, its mitotic functions are not yet fully understood. In this study, we show that TOPK plays a global mitotic role by simultaneously regulating hundreds of DNA binding proteins. C2H2 zinc finger proteins (ZFPs) constitute the largest family of human proteins. All C2H2 ZFPs contain a highly conserved linker sequence joining their multi-zinc finger domains. We have previously shown that phosphorylation of this conserved motif serves as a global mechanism for the coordinate dissociation of C2H2 ZFPs from condensing chromatin, during mitosis. Here, using a panel of kinase inhibitors, we identified K252a as a potent inhibitor of mitotic ZFP linker phosphorylation. We generated a biotinylated form of K252a and used it to purify candidate kinases. From these candidates we identified TOPK/PBK, *in vitro* and *in vivo,* as the master ZFP linker kinase. Furthermore, we show precise temporal correlation between TOPK activating phosphorylation by Cdk1 and linker phosphorylation in mitosis. The identification of this fundamental role of TOPK underscores its significance as a promising novel target of cancer therapeutics.

## INTRODUCTION

Successful completion of mitotic cell division requires the coordinated and synchronized execution of numerous biochemical and morphological events. It is now well established that phosphorylation signaling pathways play a leading role in choreographing the entry into, and exit from, mitosis [1, 2]. Traditionally, the Cdk1 kinase has been regarded as the master regulator of cell division; however, over the past two decades many other kinases have also been shown to play major mitotic roles, like Polo-like and Aurora kinases [3]. Inhibition of these kinases is widely investigated as a main approach for targeted cancer therapies [4]. Understanding the roles of additional kinases that could be essential for the integrity of mitosis will offer new promising therapeutic targets.

Much of mitosis-focused research aims at understanding the specific mechanisms and signaling pathways regulating the fidelity of chromosome segregation. However, another important aspect of mitosis involves the regulation of the DNA-binding proteins, their dissociation from DNA, distribution to daughter cells, and re-association with DNA at the M/G1 transition [5].

C2H2 zinc finger proteins (ZFPs) represent the largest family of proteins in the human proteome, comprising hundreds of members involved in almost every aspect of cell biology [6]. The several hundred members of this family include well-studied and characterized proteins like YY1, Sp1, CTCF, Ikaros, Aiolos, and Bcl6, but also dozens of proteins of yet unidentified functions. The common feature of all of these proteins, making them a family, is a zinc finger motif which mediates their sequence-specific DNA binding activity [7–9]. A ZFP can contain up to dozens of tandemly clustered zinc fingers, each having a specific DNA consensus binding site. However, efficient and stable ZFP binding to DNA is only achieved after multiple zinc fingers wrap around the double helix in a locking position [9]. Research in the 1990s provided valuable insights into the significance of small 5–7 amino acid linker sequences that join adjacent zinc fingers. Although linkers do not make any specific and direct contact with DNA base pairs, these structural motifs play a critical role in the wrapping of the ZFP around the DNA and the locking position needed for stable binding [8–14].

This fundamental function of linker peptides is reflected by a remarkably high conservation among the various C2H2 ZFPs. With an amino acid sequence resembling TGEKP [9], linker peptides have more than five thousand occurrences in the human proteome, making this motif one of the most conserved amino acid sequences in nature [9, 15].

Interestingly, the vast majority of linker peptides contain a serine or threonine residue in the first position. Phosphorylation of these residues can greatly reduce the DNA binding activity [16]. In 2002, Dovat et al found that linker peptides of two transcription factors belonging to the C2H2 ZFP family (Ikaros and Sp1) are highly phosphorylated at these residues during mitosis [17]. This phosphorylation led to the inactivation of Ikaros and Sp1's DNA binding activity. Because of the high conservation of linker peptides, the authors proposed that their phosphorylation could be a common mitotic mechanism for the global reduction of DNA binding affinity of the entire family of C2H2 ZFPs. The authors also reported that the major mitotic kinases could not phosphorylate linker sequences, and the linker kinase was not identified [17].

Our previous work on the transcription factor YY1 (also a member the C2H2 ZFP family) showed similar mitotic phosphorylation of its linker peptides [18]. This fits well with the common mechanism hypothesis. To provide evidence for this global pathway, we previously generated an antibody raised against the phosphorylated form of the most common linker peptide (TGEKP). This exact sequence accounts for about 50% of all linkers and is present in 80% of C2H2 ZFP. Using this antibody we characterized the phosphorylation of ZFP linker peptides as a highly coordinated event that starts in mid-to-late prophase and is gradually reversed during telophase [19].

However, the kinase(s) and signaling pathways involved in this mechanism remained unknown. In this study, we follow a biochemical approach to isolate and identify TOPK as the main linker kinase.

TOPK/PBK is a serine/threonine kinase associated with highly proliferating tissues [20, 21]. Over the past ten years, multiple studies have shown that the expression of TOPK is upregulated in many types of cancers and is associated with poor patient prognosis [22–28]. TOPK is known to be important for mitotic division [24, 29, 30] and that phosphorylation by Cdk1 is needed for its activation [21]. However, the main mitotic substrates of TOPK and its functional relevance to cell division are not fully understood. The results presented in this report reveal a fundamental mitotic role for TOPK in regulating the removal of hundreds of proteins from the condensing chromatin. Because TOPK is proposed to be a promising novel target in cancer therapeutics, a better understanding of its roles is of crucial significance.

## RESULTS

### Efficient inhibition of C2H2 linker kinase by K252a

**Figure 1:**
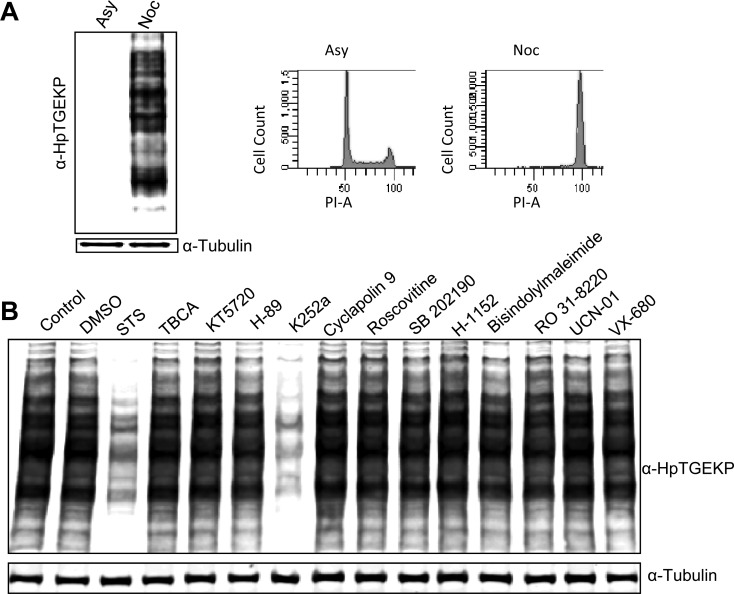
K252a can inhibit linker phosphorylation in mitotic HeLa cells **(A)** Western blot analysis of whole protein extracts from HeLa cells, growing asynchronously (Asy) or arrested with nocodazole (Noc) for 18 hours. The blot was probed with anti-HpTGEKP antibody to visualize the C2H2 ZFP linker phosphor-bands. The blot was then probed with anti-tubulin to show equal loading. A fraction of the cells were tested with FACS analysis for their cell cycle distribution, as a control (right panels) **(B)** HeLa cells arrested with nocodazole for 18 hours were collected, divided into equal fractions and treated with small-molecule inhibitors, as indicated on the blot, at 1 μM concentration for 10 minutes. Cell lysates were analyzed by Western blotting and probed with anti-HpTGEKP antibody, then with anti-tubulin antibody as loading control.

In a previous report, we characterized a global phosphorylation event occurring on the C2H2 ZFP linker peptides during mitosis. To study the simultaneous phosphorylation of hundreds of proteins, we developed a phospho-specific antibody (anti-HpTGEKP) that can accurately recognize the phosphorylated form of the consensus linker motif [19]. Using this antibody we discovered a tightly synchronized wave of phosphorylation that appears in mid-prophase and is reversed in telophase of mitosis. This global phosphorylation can be seen in Figure 1A showing a Western blot analysis of whole cell extracts prepared from HeLa cells, growing asynchronously or blocked with nocodazole (prometaphase of mitosis). Probing these extracts with anti-HpTGEKP antibody demonstrates the high abundance this phosphorylated motif very specifically in mitotic cells. The specificity of this antibody to mitotic linker phosphorylation has been previously shown [19]. Because this is a major regulatory pathway, we wanted to identify the kinase(s) that can simultaneously phosphorylate all these proteins. First, we analyzed the amino acid linker sequences from various C2H2 ZFPs with multiple phosphoprediction tools. However, the analysis did not retrieve any strong hits, and the linkers did not fit the consensus sequence of any of the well characterized kinases.

To gain insight into the identity of the linker kinase, we treated HeLa cells (blocked in mitosis with nocodazole) with a panel of small-molecule kinase inhibitors. The inhibitors chosen for this screening covered a wide range of target kinases and included molecules with varying target specificity. For example, staurosporine (STS) is a pan inhibitor with hundreds of target kinases, while K252a and RO-31-8220 are broad range inhibitors with dozens of targets. In addition, specific inhibitors for the major mitotic kinases were also included in this panel, like roscovitine (for Cdk1), VX-680 (for Aurora kinases), and Cyclapolin 9 (for Plk1). We also tested specific inhibitors for kinases with consensus sequences somewhat resembling linker peptides, like TBCA (for CKII), KT5720 (for PKA), UCN01 (for PKCs), KN62 (for CAMKII), and rapamycin (mTOR). The complete list of inhibitors and their target kinases is shown in Table S1. Interestingly, from all the inhibitors tested only staurosporine and K252a showed efficient and rapid inhibition of linker phosphorylation (Fig. 1B and Fig. S1). STS is a pan inhibitor and its effect here was not surprising; however, the inhibitory effect of K252a was very interesting, and could be used to provide leads to the identity of the linker kinase.

To ensure that the loss of linker phosphorylation in the K252a treated mitotic cells is not resulting from exiting mitosis, we assessed the phosphorylation of histone 3 Serine 10 (pH3S10), by Western blotting and immunostaining of mitotic cells treated with K252a. pH3S10 phosphorylation is the most widely used and established mitotic marker [31, 32]. Whereas linker phosphorylation was greatly reduced by treatment with K252a, the pH3S10 signal was not affected (Fig. S2A, S2B). Moreover, microscopic examination of treated cells did not show any decondensation of chromosomes, as visualized with DAPI staining (Fig. S2C).

The functional significance of linker phosphorylation in mitosis is the reduction of DNA binding activity. K252a caused the inhibition of linker phosphorylation, so it should also enhance the binding activity of C2H2 proteins, even in mitotic extracts. Therefore, we examined the binding activity of two well-characterized C2H2 ZPF, YY1 and Sp1 in protein extracts prepared from HeLa cells. Both proteins have been shown to be mitotically phosphorylated at their linker sequences [17, 18]. Analysis of their DNA binding activity in an *in vitro* electrophoretic-mobility shift assay (EMSA) showed significant reduction in protein extracts prepared from mitotic cells in comparison to extracts prepared from asynchronously growing cells, as expected. Treatment of mitotic cells with K252a prior to protein extraction resulted in a significant restoration of DNA binding activity of YY1 and Sp1 (Fig. S2D).

**Figure 2:**
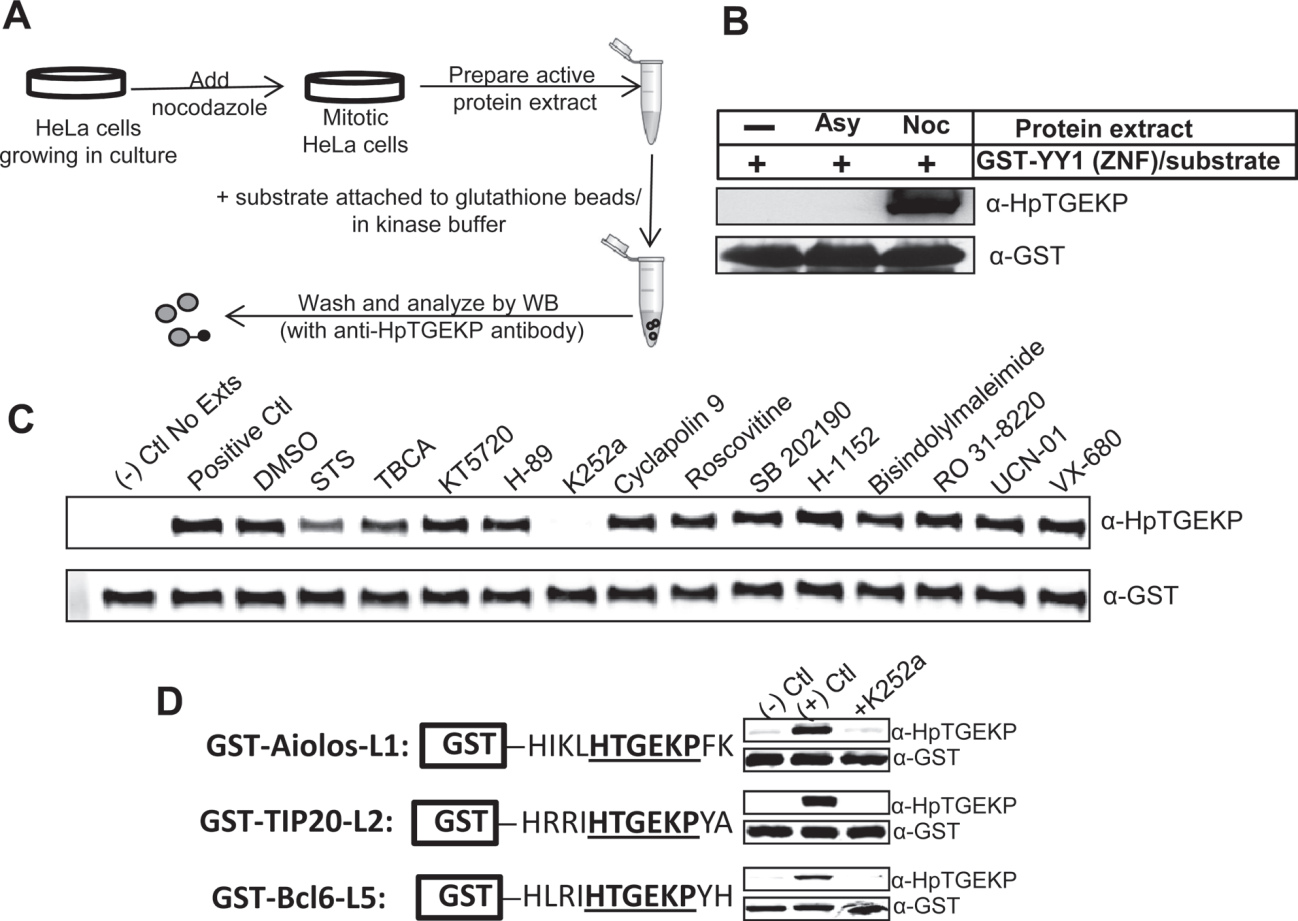
K252a can inhibit the linker kinase activity in mitotic extracts *in vitro* **(A)** Experimental outline for *in vitro* kinase assays using active mitotic protein extracts. **(B)** Western blot analysis of *in vitro* kinase assay performed as described in (A) using GST-YY1 (ZNF) as substrate coupled to glutathione beads. The blot was probed with anti-HpTGEKP antibody to show phosphorylation by mitotic extracts and anti-GST antibody to show equal substrate loading. **(C)** Protein extracts from nocodazole-arrested HeLa cells were tested in an *in vitro* kinase assay as described in (A) and (B) in the absence or presence of the indicated small molecule inhibitors. **(D)** The mitotic protein extracts were further tested in *in vitro* kinase assays with three GST-tagged linker sequences from three different proteins (as indicated), coupled to glutathione beads. The assays were performed in the absence or presence of K252a. The Western blots were analyzed by anti-HpTGEKP antibody, then with anti-GST antibody to show equal substrate loading.

Next we wanted to assess the effect of K252a on the linker kinase activity in an *in vitro* kinase assay. For this purpose, we prepared protein extracts from nocodazole-arrested HeLa cells (Fig. 2A) and tested the kinase activity of these extracts against the bacterially expressed GST-tagged DNA binding domain of the YY1 protein. As shown in Figure 2B, the mitotic extracts, but not the asynchronous extracts, efficiently phosphorylated the linker peptide of YY1. Incubation of the mitotic extracts with the small-molecule inhibitors showed again that only K252a efficiently inhibits the linker phosphorylation (Fig. 2C and Fig. S3).

This is a global mechanism occurring on many proteins; we wanted to test if K252a can inhibit the *in vitro* phosphorylation of linker peptides from proteins other than YY1. Ailos, TIP20, and Bcl6 are three transcription factors that belong to the C2H2 ZFP family. The linker peptides of these proteins have been found to be phosphorylated *in vivo* by large-scale mass spectrometry analyses [33]. We fused 12 amino acid sequences comprising linker peptides from these three ZFPs to a GST tag for bacterial expression and purification. As shown in Figure 2D, HeLa mitotic extracts efficiently phosphorylated these linker peptides in an *in vitro* kinase assay. Importantly, the addition of K252a inhibited most of the phosphorylation activity on all three linker peptides (Fig. 2D).

### Purification of the linker kinase using biotin-K252a

K252a is a derivative compound of STS that has a significantly narrower specificity range than STS. Although K252a is best known for its potent inhibition of the tyrosine receptors kinases (TrkA, B, and C), it has also been shown to inhibit many other kinases like PKA, PKC, PKG, CAMK, and kinases of the MAPK pathway [34–40]. Moreover, many kinases were found to be associated with K252a when coupled to beads in pull-down assays from cell extracts [41].

**Figure 3:**
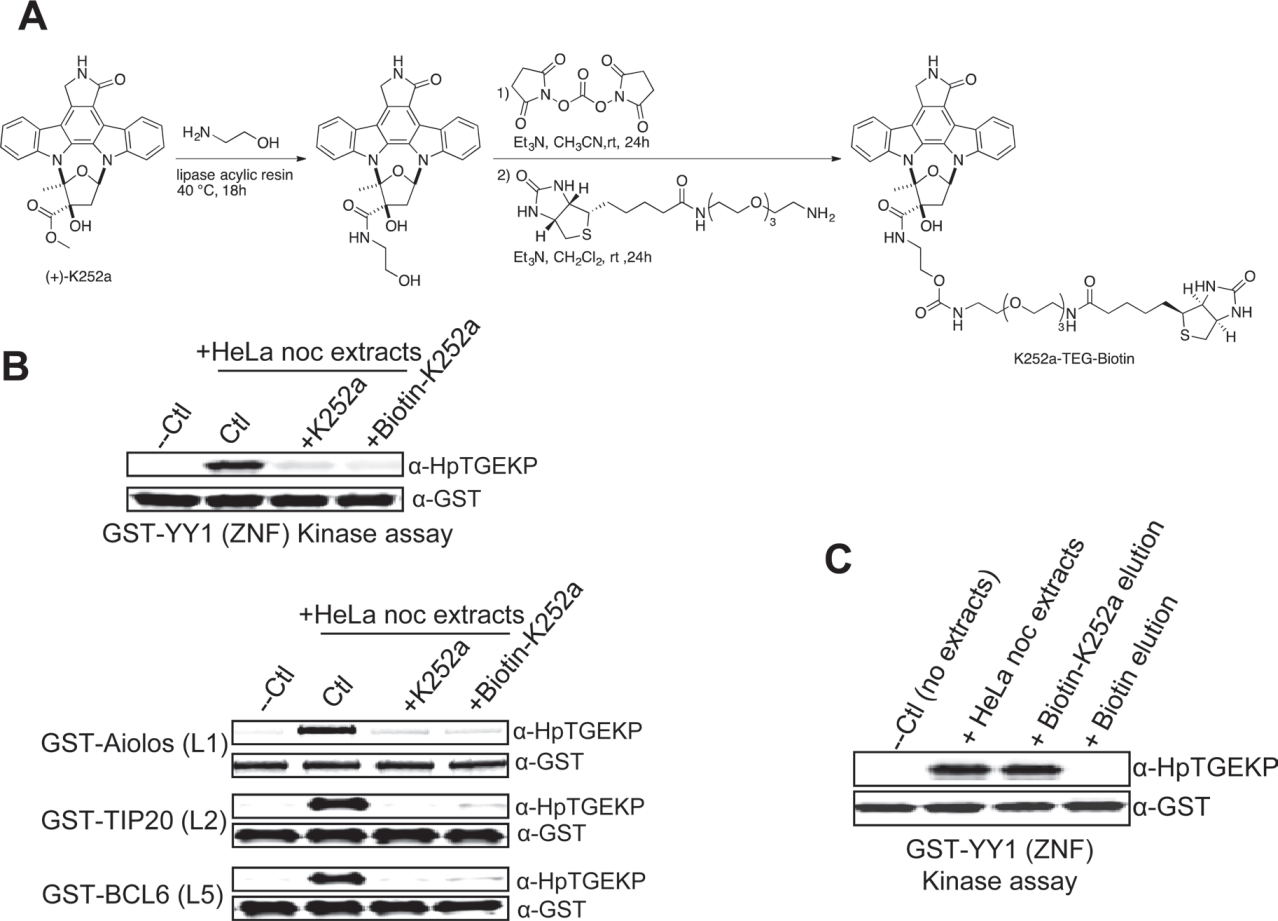
Biotinylated K252a maintains linker kinase inhibitory activity **(A)** Outline of the preparation of biotin-K252a compound. **(B)** Western Blot analysis of the *in vitro* kinase assays testing the activity of protein extracts from nocodazole-arrested HeLa cells on GST-tagged linker sequences, coupled to glutathione beads, in the absence or presence of K252a or biotin-K252a. **(C)** Biotin-K252a (or biotin as negative control) was incubated with protein extracts from nocodazole-arrested HeLa cells, followed by incubation with avidin beads. After collecting the beads by centrifugation and washing, the protein fraction bound to biotin-K252a was eluted with ATP, and tested for linker kinase activity in an *in vitro* kinase assay with GST-YY1(ZNF) as substrate. The kinase reactions were analyzed by Western blotting with anti-HpTGEKP and anti-GST antibodies. Biotin pull-down served as negative control and the total mitotic protein extracts as positive control.

The linker kinase appears to be selectively active in the short time frame of mitosis. It is likely that it has not been previously recognized as one of the K252a targets. So, we sought to purify the linker kinase based on its interaction with K252a from the active extracts of mitotic cells. For this purpose, we generated a biotinylated form of K252a that can be isolated using the biotin-avidin purification system (Fig. 3A). To ensure that the biotin-K252a compound maintains its inhibitory effects on the linker kinase, we tested it in an *in vitro* kinase assay in parallel with the parent compound. As shown in Figure 3B, the biotinylation of K252a did not affect its ability to inhibit the linker kinase activity of mitotic extracts on the DNA binding domain of YY1 (Fig. 3B upper panel), nor on the GST-fused linkers sequences of Aiolos, TIP20, and Bcl6 (Fig. 3B lower panels).

To isolate the linker kinase, we prepared total protein extracts from mitotic HeLa cells arrested by nocodazole. We incubated the extracts with biotin-K252a, or biotin as negative control. Then, we added avidin beads to pull down the biotin-K252a associated proteins. After extensive washing, we eluted the proteins by competition with high concentration of ATP. To check if the elutions contained kinase activity we tested them in an *in vitro* kinase assay. As shown in Figure 3C the protein fraction isolated with biotin-K252a contained linker kinase activity, whereas no activity was detected in the biotin control elution. The biotin-K252a elution was also able to phosphorylate the linkers of Aiolos, TIP20, and Bcl6 (not shown). Therefore, this method can enable the isolation of a protein fraction containing kinase activity for further identification.

### Identification of TOPK as a candidate linker kinase

**Figure 4:**
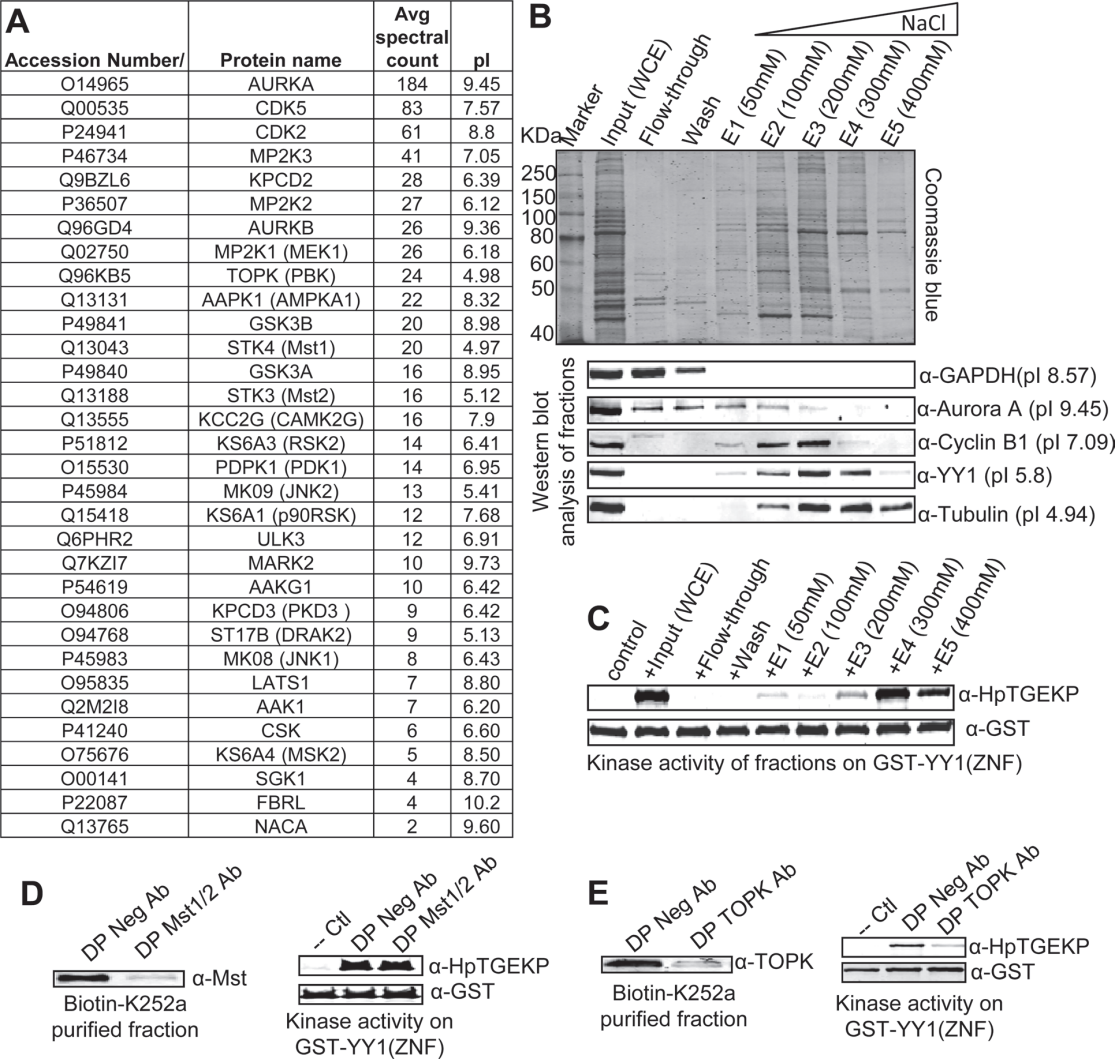
Identification of TOPK as a primary linker kinase candidate **(A)** List of the kinases physically associating with biotin-K252a (but not biotin only) in the avidin pull-down assay, as identified by mass spectrometry analysis. The kinases are listed according to the average spectral counts from the three independent pull-downs. The list of the respective isoelectric point is also included for each kinase. **(B)** Step-wise anion exchange fractionation of protein extracts from nocodazole-arrested HeLa cells. The proteins were bound to the resin of a spin column and then eluted at the indicated NaCl concentrations. Samples from the input extract, wash, and fractions were run on SDS-PAGE gels and either stained with Commassie blue or Western blotted and probed with the indicated antibodies. **(C)** Samples were also tested in *in vitro* kinase assay with GST-YY1(ZNF) coupled to beads, as substrate. The kinase reactions were Western blotted and probed with anti-HpTGEK and anti-GST antibodies (please also refer to Figure S4). **(D–E)** An elution fraction of a biotin-K252a pull-down was immunodepleted overnight with anti-Mst1/2 antibody or anti-TOPK antibody; the negative control depletion was incubated with a negative rabbit antibody (D and E left panels). The Mst1/2 or TOPK immunodepleted biotin-K252a fractions were tested in *in vitro* kinase assays with GST-YY1(ZNF) coupled to beads. The kinase reactions were Western blotted and probed with anti-HpTGEKP and anti-GST antibodies (D and E right panels).

To identify the mitotic proteins that can bind to biotin-K252a, we performed pull-down experiments (as described above) from three independently prepared HeLa mitotic extracts. Small fractions of the beads were incubated with ATP and the elutions were tested in kinase assays, verifying that the linker kinase is in the biotin-K252a, but not biotin only, pull-downs (not shown). The remainder of the beads were subjected to tryptic digests and analyzed on an Orbitrap mass spectrometer. Because many proteins can associate non-specifically with avidin beads, we only considered the proteins identified in the biotin-K252a pull down, but not in the biotin negative control. The identified proteins are shown in Figure 4A. As expected, there were a significant number of kinases associated with biotin-K252a, including a number of previously known targets [41].

To identify candidates for further testing, we sought to gain more insights into the physical characteristics of the linker kinase. For this, we fractionated a HeLa mitotic extract preparation on an anion exchange spin column. By increasing step wise the salt concentration, we obtained multiple protein fractions of varying affinities to the exchange column. The binding affinity of proteins to an anion exchange column is an indication of their net charge and isoelectric points. To check the separation of proteins, we analyzed the elution fractions by Western blotting and probed with antibodies for proteins of known isoelectric points, as markers of fractionation (Fig. 4B). On the other hand, we tested the fractions in an *in vitro* kinase assay (Fig. 4C and Fig. S4). A comparison of the kinase activity of the fractions along with probing for the known markers indicated that the linker kinase has an isoelectric point of less than 5. Although this is not a precise analysis, it is accurate enough to select candidates for further testing. Screening the isoelectric points of the proteins identified by mass spectrometry showed that Mst1/2 and TOPK are possible candidates. To check if these kinases are responsible for the linker kinase activity in the biotin-K252a elution fraction, we immunodepleted the putative candidates using their respective specific antibodies. Whereas the immunodepletion of Mst 1/2 had no effect on the kinase activity of the fraction, depletion of TOPK greatly reduced this activity (Fig. 4D–4E). Furthermore, we confirmed the enrichment of TOPK in the anion exchange fractions containing linker kinase activity (Fig. S5). This indicates that TOPK is a strong candidate.

### Activated recombinant GST-TOPK can phosphorylate C2H2 ZFP linkers *in vitro*

**Figure 5:**
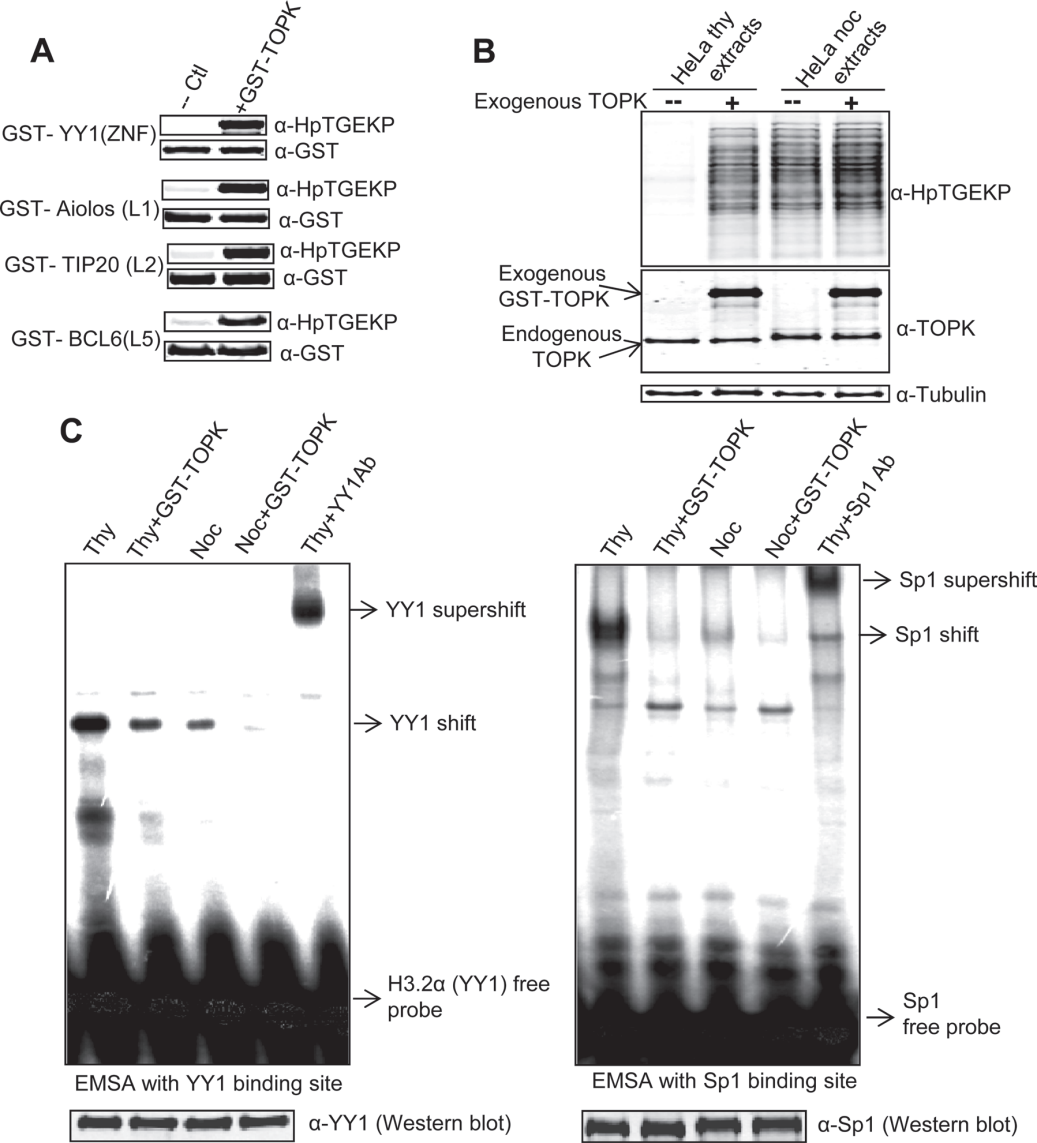
TOPK can phosphorylate C2H2 linker sequences *in vitro* **(A)** Purified/recombinant active GST-TOPK was incubated in kinase buffer with purified GST-tagged: YY1(ZNF), Aiolos (L1), TIP20(L2), or Bcl6(L5). The kinase reactions were analyzed by Western blotting with anti-HpTGEKP and anti-GST antibodies. **(B)** Whole cell extracts prepared from HeLa cells arrested in S-phase (by thymidine) or mitosis (by nocodazole) were incubated in kinase buffer with or without purified/recombinant active GST-TOPK. The kinase reactions were analyzed by Western blotting with anti-HpTGEKP, anti-TOPK, and anti-Tubulin antibodies. **(C)** EMSA testing of the kinase reactions analyzed in (B) with radioactively labeled double-stranded DNA oligonucleotides comprising the consensus binding sites for YY1 and Sp1. The specificity of the YY1 and Sp1 shifts were confirmed by supershifting with their respective antibodies. The kinase reactions were also Western blotted and probed with YY1 and Sp1 antibodies to confirm the equal levels of the proteins in all reactions and that no degradation occurred during the kinase reaction.

To test TOPK for ZFP linker kinase activity, we incubated activated purified GST-TOPK with purified GST-fusion linker peptides in an *in vitro* kinase assay. As shown in Figure 5A, GST-TOPK efficiently phosphorylated the linker sequences from YY1, Aiolos, TIP20, and Bcl6. Linker peptides are present in hundreds of proteins. The best approach to cover the largest number of proteins possible in one assay, is to use total cell protein extract as a substrate in an *in vitro* kinase assay with GST-TOPK. For this, we prepared protein extracts from HeLa cells arrested in S-phase (by thymidine) or mitosis (by nocodazole). The synchrony of the cells was assessed by FACS analysis (not shown). The protein extracts were incubated in kinase buffer in the presence of ATP, with or without purified active GST-TOPK. We have previously shown that the linker phosphorylation is very specific to mitotic cells and that S-phase proteins are devoid of this phosphorylation [19]. As expected, the thymidine extracts did not show any linker phosphorylation, whereas the nocodazole extracts contained a high abundance of phospho-proteins. Interestingly, addition of activated recombinant GST-TOPK to the thymidine extracts resulted in a phosphorylation signal comparable to that of nocodazole extracts (Fig. 5B). This shows that TOPK can phosphorylate linker sequences on a very large number of proteins. There was only a slight increase in the linker phosphorylation of nocodazole extracts upon the addition of GST-TOPK. This is reasonable, as the phosphorylation of mitotic proteins (especially from nocodazole-arrested cells) approaches full occupancy [42].

Because mitotic linker phosphorylation is functionally related to a reduction in DNA-binding activity of C2H2 ZFPs, we wanted to check if the GST-TOPK phosphorylation of S-phase proteins can result in similar effects. For this, we tested the binding activity of two representative ZFPs, YY1 and Sp1, in an EMSA assay. For the source of the proteins, we used the same kinase reactions shown in Figure 5B. YY1 and Sp1 in thymidine extracts bound very efficiently to their target consensus sequence, with a significant reduction of binding activity in the nocodazole extracts. This reduction of activity was similarly observed in the thymidine extracts incubated with GST-TOPK (Fig. 5C). This indicates that activated recombinant GST-TOPK can phosphorylate linker sequences and inactivate the DNA binding activity of S-phase C2H2 proteins *in vitro*.

### Spatiotemporal correlation between mitotically active TOPK and linker phosphorylation

We have previously shown that the phosphorylation of linker peptides occurs exclusively in the cellular nucleus at mid-late prophase [19]. Therefore, we wanted to check if TOPK is present in the nucleus at this stage. Immunostaining of TOPK in HeLa cells showed that it is localized both in the nucleus and the cytoplasm (Fig. S6), thereby having access to phosphorylate the nuclear linkers.

It has been previously shown that Cdk1 phosphorylates TOPK in the N-terminal domain at threonine 9 and increases its kinase activity in mitosis [21]. Using a phospho-specific antibody for TOPK threonine 9 phosphorylation (anti-pTOPK), we show that TOPK is heavily phosphorylated at this site in protein extracts prepared from nocodazole-arrested (mitotic), but not asynchronously growing, HeLa cells (Fig. S7A). The TOPK in the active anion exchange fractions and the biotin-K252a elution, in addition to the recombinant activated GST-TOPK, are phosphorylated at threonine 9 (Fig. S7B–S7E).

**Figure 6:**
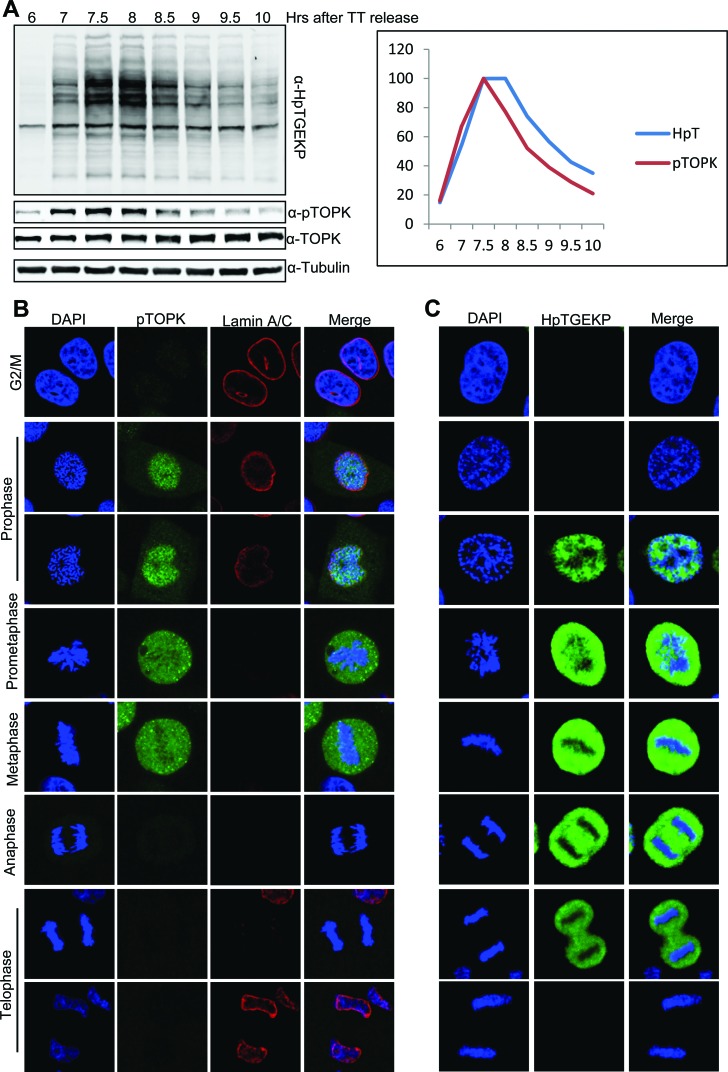
Timing and localization of mitotically active pTOPK in correlation with HpTGEKP **(A)** HeLa cells were synchronized at G1/S by double-thymidine block and then released. Cells were collected for whole cell extract preparation at 6–10 hours after the release (as indicated), as they entered and exited mitosis. Protein extracts were analyzed on a Western blot with anti-HpTGEKP, anti-TOPK, and anti-pTOPK antibodies. The blot was further probed with anti-Tubulin as a loading control. The signals from the anti-HpTGEKP and anti-pTOPK antibodies were quantified and plotted, after setting the highest signal from each antibody to 100. **(B-C)** HeLa cells grown on coverslips were synchronized with a single thymidine block and then released (right panel). Cells were fixed 7–9 hours after the release, permeabilized, and immunocytostained with anti-pTOPK, anti-Lamin A/C (to visualize the nuclear envelope) (B), and anti-HpTGEKP (C) antibodies. Cells were stained with DAPI to visualize the DNA. Please refer to Figure S6 (for total TOPK immunostaining) and Figure S8 (for pTOPK immunostaining in multiple mitotic cells in the same field).

To investigate the correlation between the activating phosphorylation of TOPK and phosphorylation of its linker substrates *in vivo*, HeLa cells were synchronized by double thymidine block and released. Whole cell extracts were prepared from cells collected at 6–10 hours after the release, as they entered and exited mitosis. Western blot analysis of these protein extracts with anti-HpTGEKP and anti-pTOPK antibodies showed a close temporal correlation in the occurrence of these phosphorylation events at the entry into mitosis. As cells exited from mitosis, TOPK was rapidly dephosphorylated before the dephosphorylation of linker peptides (Fig. 6A). To obtain higher resolution of the timing and localization of these two events, we immunocytostained and examined single HeLa cells at the different stages of mitosis. As shown in Figure 6B, the activating phosphorylation of TOPK occurs early in prophase. Interestingly, the pTOPK signal was primarily nuclear, versus the ubiquitous localization of total TOPK shown in Figure S6. This result agrees well with the fact that pTOPK phosphorylation is caused by mitotic Cdk1/cyclinB1 which is predominantly nuclear at this stage [43]. As expected [19], the nuclear linker phosphorylation occurred in mid-late prophase immediately after the TOPK mitotic phospho-activation. On the other hand, dephosphorylation of TOPK appears to occur rapidly at the metaphase-anaphase transition (Fig. 6B and Fig. S8). Again, this result conforms to what is known about Cdk1 substrates [1]. The dephosphorylation of linker peptides follows the dephosphorylation of TOPK and gradually occurs during telophase.

Taken together, these observations present a solid spatiotemporal correlation between the mitotic activation of TOPK and its proposed role as the linker kinase.

### TOPK knockdown reduces linker phosphorylation in HeLa cells

**Figure 7:**
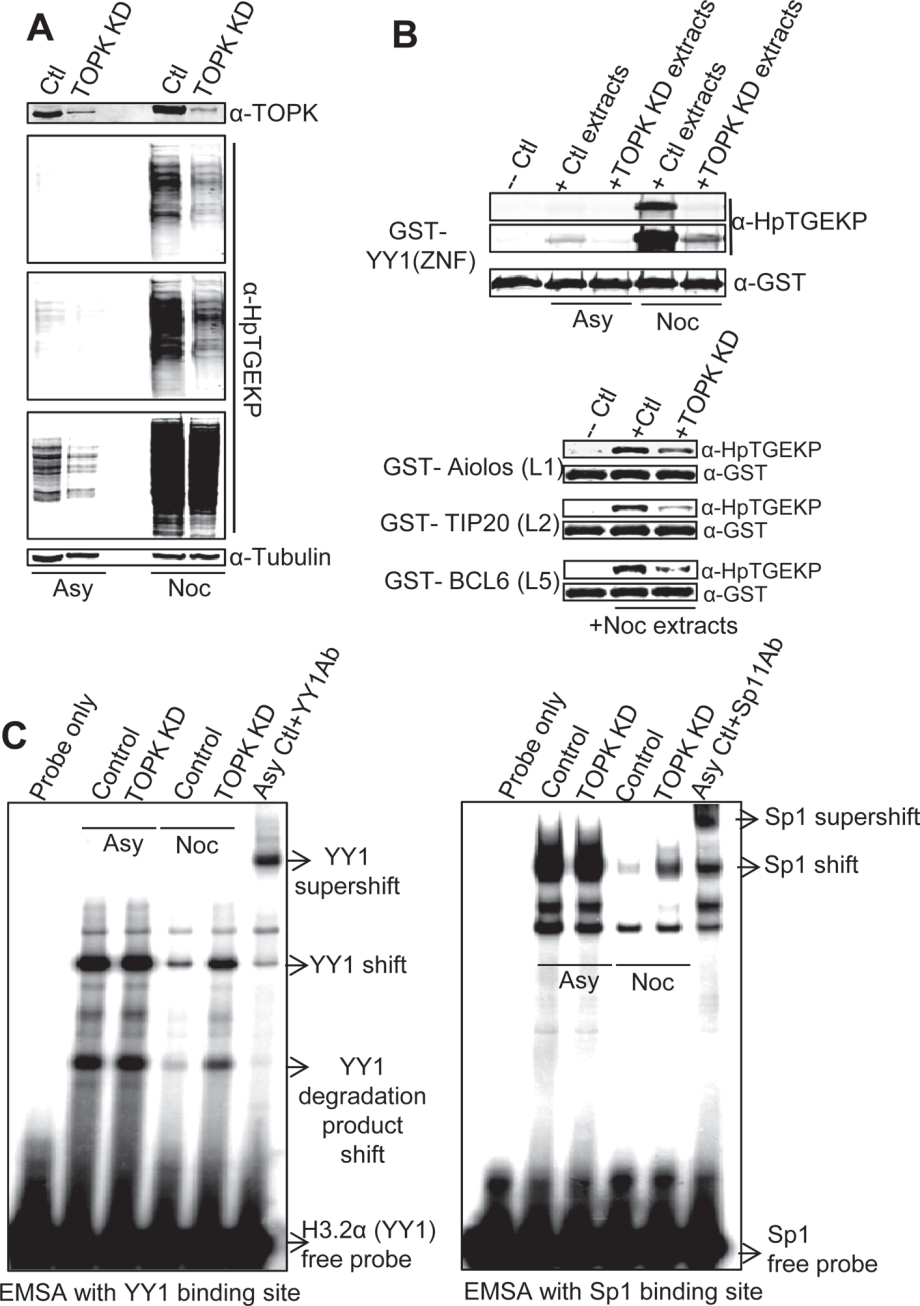
TOPK knockdown results in significant reduction of linker phosphorylation *in vivo* HeLa cells were transfected with TOPK specific siRNA, or scrambled siRNA, and either left growing asynchronously or synchronized with thymidine-nocodazole block. **(A)** Western blot analysis of WCE of siRNA transfected HeLa cells probed with anti-TOPK and anti-HpTGEKP antibodies. Three different exposures of the HpTGEKP signal are displayed to visualize the differences in HpTGEKP signal between control and TOPK knockdown samples, both in asynchronous and mitotic cells. The blot was further probed with anti-Tubulin as a loading control. **(B)** The proteins extracts probed in (A) were tested in an *in vitro* kinase assays with purified GST-tagged: YY1(ZNF), Aiolos (L1), TIP20(L2), or Bcl6(L5) as substrates. The kinase reactions were analyzed by Western blotting with anti-HpTGEKP and anti-GST antibodies. **(C)** The proteins extracts probed in (A) were tested in *in vitro* EMSA assays by incubation with radioactively-labeled double-stranded DNA oligonucleotides comprising the YY1 and Sp1 consensus binding sites. The YY1 and Sp1 specific shifts are indicated and confirmed by super-shift analysis with their respective specific antibodies.

Finally, we wanted to obtain conclusive evidence that TOPK can phosphorylate linker peptides *in vivo*. Therefore, we knocked down TOPK and analyzed the protein extracts for linker phosphorylation. In two parallel knockdown experiments, we kept one cell population growing asynchronously and arrested the second by a thymidine-nocodazole block following the knockdown. As shown in Figure 7A, knockdown of TOPK resulted in global reduction of the HpTGEKP signal, both in asynchronous and mitotic cells. Three different exposures are presented because it is difficult to assess this phosphorylation in asynchronous and mitotic cells simultaneously due to the large difference in phospho-linker abundance. Although the HpTGEKP signal was not completely abolished, there was a clear global reduction in all of the phospho-bands. We further tested the extracts in an *in vitro* kinase assay using the GST-fused linkers from YY1, Aiolos, TIP20, and Bcl6 as substrates. The protein extracts from cells with TOPK knockdown showed significant reduction of linker kinase activity (Fig. 7B). Moreover, there was a partial regain of binding affinity of YY1 and Sp1 in mitotic extracts where TOPK was knocked down (Fig. 7C). These results clearly indicate that TOPK is a global mitotic linker kinase *in vivo*.

## DISCUSSION

In this study, we report the identification of the TOPK/PBK kinase as a master mitotic regulator of C2H2 ZFPs, which represent the largest family of human proteins. We have previously shown the tight synchrony of the C2H2 ZFPs’ linker phosphorylation on hundreds of proteins from prophase to telophase, associated with their dissociation from condensed chromatin [19]. This synchrony fits well with the overall coordinated regulation of critical mitotic events.

To identify the C2H2 ZFP linker kinase, we screened the effects of multiple small molecule kinase inhibitors, using the anti-HpTGEKP phospho-specific antibody, on the linker phosphorylation in mitotic cells. After the identification of the strong inhibitory effects of K252a, we coupled K252a to a biotin tag and used this conjugate to isolate many interacting kinases, as identified by mass spectrometry. From these kinases, we found that TOPK was the most likely candidate. In an *in vitro* assay, recombinant activated GST-TOPK was able to phosphorylate several linker sequences from YY1, Aiolos, TIP20, and Bcl6. Furthermore, when we used total protein extracts from an S-phase HeLa as substrates, we found that activated GST-TOPK was able to generate a phosphorylation pattern similar to that of extracts from mitotic cells. This demonstrates the large number of C2H2 ZFP substrates of TOPK.

Importantly, knockdown of TOPK by specific siRNA resulted in a significant decrease of total linker phosphorylation *in vivo*. The phosphorylation was not entirely abolished upon TOPK knockdown. This could be due to two main non-mutually exclusive possibilities. First, the knockdown does not completely eliminate the enzyme. Second, it is likely that other related kinases could contribute, to a lesser extent, to the linker phosphorylation, especially in the absence of TOPK. However, the uniform reduction in the phosphorylation signal across the blot clearly reflects the global nature of TOPK substrates and indicates that TOPK is the major linker kinase. Moreover, *in vitro* phosphorylation of total cell proteins by TOPK greatly reduced the binding activity of two prominent C2H2 ZFPs, YY1 and Sp1 (Fig. 5C). On the other hand, *in vivo* knockdown of TOPK resulted in partial restoration of this binding activity in mitotic extracts. These results provide clear functional effects of TOPK phosphorylation of ZFP linker peptides.

The cloning of TOPK/PBK is relatively recent and its physiological functions are not yet fully understood. In the year 2000, Gaudet et al reported the cloning of a kinase that bound to the PDZ domain of the tumor suppressor hDlg protein in a yeast two-hybrid assay. The kinase was named PBK (PDZ-binding kinase) [21]. The authors analyzed a panel of normal human tissues for PBK expression and found it to be primarily expressed in the placenta. Furthermore, they showed that PBK is phosphorylated and activated by Cdk1 in mitosis. In the same year, Abe et al cloned a kinase that is upregulated in lymphokine-activated killer T cells, and called it TOPK (T-LAK cell originated protein kinase) [44]. The authors also analyzed a panel of human tissues and found TOPK to be upregulated in the testis (later shown to be primarily in the proliferating spermatocytes [45]). From these early reports, it became apparent that TOPK/PBK is associated with highly proliferative tissues and that it is involved in the mitotic process. Later studies underscored the role of TOPK in cell proliferation as the knockdown of TOPK resulted in decreased cell growth and proliferation [23, 24]. We also observed that knockdown of TOPK results in significant reduction in cell proliferation (not shown). The mitotic significance of TOPK was demonstrated as TOPK knockdown resulted in abnormal completion of mitosis [24, 29, 30].

Because of its correlation with cell proliferation, multiple studies analyzed the expression of TOPK in cancer tissues. TOPK was found to be upregulated with many types of malignancies, with higher TOPK protein levels correlating with higher aggressiveness and poor prognosis [22–28]. Importantly, the oncogenic potential of TOPK was demonstrated by its ability to transform normal mouse epidermal (JB6 Cl41) cells [27]. On the other hand, recent reports have implicated TOPK in resistance to apoptotic stimuli in cancer cells. TOPK was found to enhance the resistance of MCF-7 cells to DNA damaging agents [23] and resistance of HeLa cells to TRAIL [46]. Moreover, TOPK was shown to interact with p53 in HCT116 cells and negatively affects its activation of p21 in response to doxorubicin treatment [47]. Whether the anti-apoptotic and mitotic functions of TOPK are connected is not known. Our results show that linker phosphorylation occurs exclusively in mitotic cells under normal physiological conditions. In our *in vitro* kinase assay (Fig. 5B), endogenous TOPK from S-phase extracts was not able to phosphorylate the linker peptides present in the protein extracts. The mitotic activation of TOPK by Cdk1 could direct its activity towards a specific set of substrates. It would be interesting to test, in future studies, if interphase TOPK can phosphorylate linker sequences under stress conditions, like DNA damage and/or apoptosis.

Finally, mitosis is now known to be a critical window of preserving cell identity or for reestablishment of transcriptional programs in the transition to differentiation. This is primarily due to the effects of the global dissociation of transcription factors concomitant with the mitotic silencing of transcription [48, 49]. Understanding the mitotic mechanisms regulating transcription factors have been shown to have implications in the field of stem cell reprogramming and somatic-cell nuclear transfer (SCNT) experiments [49]. In this regard, the finding that mitotic TOPK inactivates a large group of transcription factors could have valuable implications in the field of stem cell research.

In summary, we have demonstrated that TOPK is a major mitotic kinase that can simultaneously phosphorylate hundreds of proteins within minutes in the prophase stage. This is a unique mechanism and TOPK is the first kinase to be identified as a master regulator of an entire family of transcription factors based on their conserved motif. This is particularly important because TOPK is a promising target for cancer therapy, and a just published report showed that a TOPK specific inhibitor can cause tumor regression in xenograft models of human cancers [50]. The findings we report here enhance our knowledge of the physiological function(s) of TOPK and could lead to a better understanding of its role in tumorigenesis.

## MATERIALS AND METHODS

### Cell culture and synchronization

HeLa S3 cells, originally obtained from American Type Culture Collection (ATCC), were grown at 37°C in 5% CO2 in DMEM (Cellgro) supplemented with 10% fetal bovine serum (FBS; Sigma) and 1% penicillin-streptomycin (Cellgro). Cells were arrested at pro-metaphase of mitosis by adding nocodazole (Sigma) (50 ng/ml) for 16–18 hours; cells were then collected by shake-off. To block cells in S phase, thymidine (Sigma) was added to a final concentration of 2.5 mM for 18 hours. For double-thymidine synchronization at G1/S cells were blocked for 18 h in thymidine, released for 9 h and blocked again for 17 hours, then released for time point collection. For thymidine-nocodazole synchronization, cells were blocked in thymidine for 18 hours, released for 3 hours, and then nocodazole was added for 12 hours. For small-molecule inhibitor screening, HeLa cells were arrested with nocodazole, collected by shake-off, distributed equally into wells, and incubated with the inhibitors (final concentration of 1 μM) for 10 minutes.

### Primary antibodies

Rabbit polyclonal antibodies: anti-TOPK (H-152), anti-pH3S10 (-R), anti-YY1 (H414), anti-Sp1 (PEP 2), anti-Mst1/2(anti-Krs-1/2 C-19), and mouse monoclonal antibodies: anti-TOPK (A-3), anti-Tubulin (B-7), anti-Cyclin B1 (GNS1), anti-Lamin A/C (636), and anti-GST (B-14) were purchased from Santa Cruz Biotechnology. Anti-Aurora A mouse monoclonal antibody (35C1) was purchased from Invitrogen. The rabbit polyclonal antibody anti-HpTGEKP was raised against the phospho-epitope: Ac-C(Ahx)HpTGEKP-amide and was purified at New England Peptide. The specificity of this antibody to its target linker sequence and its phosphospecifcity was previously characterized [19].

### Whole cell extract (WCE) preparation

Cells were washed with ice-cold PBS, and lysed in freshly prepared lysis buffer (50 mM Tris pH 8.0, 100 mM NaCl, 0.5% Triton-X 100, 1 mM EDTA, 10 mM NaF, 10 mM β-glycerophosphate, 10 mM sodium orthovanadate, and a cocktail of protease inhibitors), for 15 minutes on ice. The crude lysates were then cleared by centrifugation for 15 minutes at 4°C. The cleared lysates were quantified using Bradford assay (Bio-Rad). For detection of the pH3S10 signal, a fraction of the cells was immediately mixed with 2X Laemmli buffer and boiled, and then cleared by centrifugation. The loading volumes for pH3S10 probing were calculated according to the quantification of the corresponding clear lysates. Equal loading was further confirmed by anti-tubulin probing.

### Western blotting

Proteins were separated on a SDS-PAGE gel and transferred to a nitrocellulose membrane. After transfer, the membrane was blocked in TBSTM (Tris-buffered saline, 0.5% Tween20, 5% Milk) for 30 minutes. Probing with the indicated primary antibodies in blocking solution was for 2 hours at room temperature (RT) or overnight at 4°C. Donkey anti-mouse (IRDye 680LT) or anti-rabbit (IRDye 800CW) secondary antibodies (LiCOR) were added for 1 hour at RT. Blots were imaged using the LiCOR Odyssey system.

### Indirect immunofluorescence

Cells seeded on coverslips were grown and synchronized as mentioned above and indicated in the figures. For immunostaining, cells were fixed with 3.7% formaldehyde for 10 minutes at RT, followed by permeabilization for 10 minutes with 0.2% Triton-X 100. Cells were blocked in TBST (with 3% IgG-free BSA) for an hour, then incubated with the indicated primary antibodies for 2 hour at RT. Anti-mouse and anti-rabbit secondary antibodies, conjugated to Alexa-Fluor 488, 546, and 647 dyes, were purchased from Molecular Probes. DNA was stained with DAPI (2 μg/ml). Images were captured using a confocal fluorescent microscope (Leica micro-systems).

### Flow cytometry

For cell cycle analysis based on DNA content, cells were fixed with 70% ethanol, washed and resuspended in propidium iodide (PI) solution (50 μg/ml PI, 200 μg/ml RNase A, 0.1% Triton-X 100 in PBS) and incubated for 30 min at 30°C. Cells were analyzed on a fluorescence-activated cell sorter (FACS; FACS Canto; Becton Dickinson), and images were generated using BD FACS Diva software.

### Electrophoretic mobility shift assays (EMSA)

Double-stranded DNA oligonucleotides (Integrated DNA Technologies) were end-labeled using T4-polynucleotide kinase (New England BioLabs) and ^32^P gamma-ATP (Perkin-Elmer). EMSA conditions were as previously described [51]. The dry gels were exposed to a phosphorimager screen and scanned using a Typhoon 9410 Imager (Amersham Biosciences). The DNA sequences of the oligos used were: H3.2α [51]: 5′-GATCCTCGGCCGTCATGGCGCTGCAGGAGGCA-3′ (YY1 binding site) and Sp1 consensus binding site [52]: 5′-ATTCGATCGGGGCGGGGCGAGC-3′. To super-shift specific binding complexes anti-YY1 or anti-Sp1 antibodies were incubated with the binding reaction mixture.

### *In vitro* kinase assays

Kinase reactions were performed in kinase buffer (50 mM Tris pH 7.4, 10 mM MgCl_2_, 2 mM ATP, 20 mM beta-glycerophosphate, 20 mM NaF, 20 mM sodium orthovanadate) for 1 hour at 30°C, with shaking. The GST-tagged substrates were immobilized on glutathione beads (Pierce). For kinase activity, either HeLa whole cell extract (40 μg) or activated/purified GST-TOPK (SignalChem) were incubated with the beads. After completion, the reactions were centrifuged briefly, the reaction mixture was aspirated and the beads were washed twice with TBST, then 2X Laemmli buffer was added and the reactions were boiled for 5 minutes. After separation on the SDS-PAGE gel, proteins were transferred to a nitrocellulose membrane and probed with the indicated antibodies. For using HeLa total protein extracts as substrates (Fig. 5B), whole cell extracts (30 μg of total protein) were incubated in solution with purified active TOPK, a fraction of the reaction was immediately tested in EMSA assays (after completion of the kinase reaction), and the other fraction was mixed with 2X Laemmli buffer and processed for Western blotting analysis.

### Bacterial plasmids

The pGEX-2T-YY1 (ZNF) plasmid containing the coding region of the DNA binding domain of YY1 (amino acids 273–414) was a kind gift from Dr. Bernhard Lüscher (Aachen University, Germany). To generate the GST-fused linker peptides, synthetic double-stranded DNA oligonucleotide (Integrated DNA Technologies) were sublconed into the BamHI/EcoRI cloning site of pGEX-2T vector. The nucleotide sequences of the oligos used are:

For the Aiolos linker 1 sequence: 5′GATCCCACATT AAACTGCACACAGGGGAAAAACCTT TTAAG-3′
For the TIP20 linker 2 sequence: 5′-GATCCCACCGG CGCATCCACACGGGCGAGAAGCCCTA CGCC-3′
For the Bcl6 linker 5 sequence: 5′-GATCCACCTG CGAATCCACACAGGAGAGAAACCTTACCAT-3′

### Bacterial expression and purification GST-tagged

Rosetta (DE3) cells (Novagen) were transformed with the pGEX-2T fusion constructs and grown overnight in LB Miller broth medium (EMD) with ampicillin (100 μg/ml final concentration). The overnight culture was diluted 1:10 in the same medium (with ampicillin) and grown to a density of 0.6 O.D. (about 1 hour), then induced with isopropyl β-D-1-thiogalactopyranoside (IPTG-Sigma-Aldrich) at a final concentration of 0.5 mM for about 4 hours. Cells were pelleted by centrifugation and then resuspended in lysis buffer (ice-cold phosphate-buffered saline (PBS) pH 8.0 or 50 mM Tris pH 8.0, 150 mM NaCl) supplemented with a cocktail of protease inhibitors (Sigma). The suspension was sonicated on ice (three bursts, 15 seconds each, with 2 minutes intervals between sonication bursts to allow cooling). Lysates were cleared by centrifugation, and then incubated with glutathione beads (Pierce) with rocking for 2–4 hours at 4°C. The resulting slurry of beads and lysates was centrifuged at 500xg for 2 minutes at 4°C. The beads were washed 3 times with lysis buffer.

### Preparation of biotin-TEG-K252a

#### (+)-K252a alcohol derivative

The preparation of (+)-K252a alcohol derivative was based on a procedure reported by Ong et al. [41] To a solution of (+)-K252a (50 mg, 0.11 mmol) in ethanolamine (10 mL, 160 mmol) was added lipase acrylic resin from *Candida antarctica* at room temperature. The reaction mixture was stirred at 40**°**C for 18 h, then filtered, washed with MeOH, and concentrated under reduced pressure. Saturated NH_4_Cl was added to the residual crude oil, and the resulting mixture was then extracted with EtOAc (3x). The combined organic layer was washed with brine, dried over MgSO_4_, and concentrated under reduced pressure to give a crude mixture, which was purified by flash chromatography on silica gel (1 to 5% MeOH/CHCl_3_) to afford 31 mg (96% yield) of pure product. Characterization data matched what was reported by Ong et al. ^1^H HNMR (600 MHz, Acetone-d6) δ 9.34 (d, *J* = 7.9 Hz, 1H), 8.09–8.02 (m, 3H), 7.81 (d, *J* = 8.2 Hz, 1H), 7.57 (br, 1H), 7.49 (t, *J* = 7.6 Hz, 1H), 7.45 (t, *J* = 7.8 Hz, 1H), 7.35 (d, *J* = 7.5 Hz, 1H), 7.27 (d, *J* = 7.5 Hz, 1H), 7.06 (dd, *J* = 7.6, 4.8 Hz, 1H), 5.06 (d, *J* = 5.3 Hz, 2H), 3.79 (d, *J* = 5.8 Hz, 2H), 3.62-3.51 (m, 2H), 3.42 (dd, *J* = 13.8, 7.6 Hz, 1H), 2.42 (dd, *J* = 13.8, 4.8 Hz, 1H), 2.26 (s, 3H).

#### K252a alcohol NHS derivative

To a solution of (+)-K252a alcohol derivative (4.6 mg, 9.3 μmol) in dry CH_3_CN (926 μL, 10 mM) were added *N,N’*-disuccinimidyl carbonate (3.6 mg, 14 μmol) and TEA (2.6 μL, 19 μmol). The reaction mixture was stirred at room temperature for 24 h. The solvent was then removed under reduced pressure and the crude product was directly purified by column chromatography on silica gel (5 to 10% MeOH/CHCl_3_) to afford 3.1 mg (53%) of product, which was carried forward immediately to the next step.

#### K252a-TEG-Biotin

To a solution of K252a alcohol NHS derivative (3.1 mg, 4.9 μmol) in dry DCM (486 μL, 10 mM) were added (+)-biotinyl-3, 6, 9-trioxaundecanediamine (2.1 mg 5.1μmol) and TEA (1.4 μL, 9.7 μmol). The reaction mixture was stirred at room temperature for 24 h. The solvent was then removed under reduced pressure and the crude product was directly purified by column chromatography on silica gel (5 to 10% MeOH/CHCl_3_) to afford 2.0 mg (44%) of product.

#### Pull down with biotin-K252a

Mitotic protein extracts (10 mgs total protein) from nocodazole-arrested HeLa cells were incubated with biotin-K252 (1 μM final concentration) rotating at 4°C for 20 minutes. Biotin was incubated in the negative control pull-down reaction. Then, avidin agarose (Pierce) was added to the extracts and the mixture was incubated for another 20 minutes rotating at 4°C. The beads complex was then centrifuged at 1000 xg for 1 minute, and then washed 5 times with lysis buffer. The pulled-down proteins were then eluted with lysis buffer containing 50 mM ATP. The elutions were then tested in *in vitro* kinase assays. For the immunodepletion of Mst1/2 and TOPK, a biotin-K252a elution fraction was incubated with anti-Mst1/2 or TOPK antibodies overnight at 4°C; then, protein A/G beads (Santa Cruz Biotechnology) were added for an hour. After centrifugation, the supernatants were collected; a sample was analyzed in a Western blot with anti-Mst1/2 and anti-TOPK antibodies to assess the depletion; another sample was tested in an *in vitro* kinase reaction.

#### Mass spectrometry analysis of biotin-K252a associated proteins

Pull-down experiments were performed, as described above, from three independent mitotic protein extracts from nocodazole-arrested HeLa cells. A fraction of the beads was eluted with ATP and tested as described above. The rest of the beads were subjected to tryptic digest and analyzed by Mass spectrometry. An externally calibrated Thermo LTQ Orbitrap Velos nLC-ESI-LIT-Orbitrap (high-resolution electrospray tandem mass spectrometer) was used with the following parameters: nLC-MS/MS was run in technical triplicate to enable normalization and analysis. A 2cm, 100 μm i.d. trap column (SC001 Easy Column from Thermo-scientific) was followed by a 10cm analytical column of 75 μm i.d. (SC200 Easy Column from Thermo-scientific). Both trap column and analytical column had C18-AQ packaging. Separation was carried out using Easy nanoLC II (Thermo-Scientific) with a continuous, vented column configuration. A 2 μL sample was aspirated into a 20 μL loop and loaded onto the trap. The flow rate was set to 300 nL/min for separation on the analytical column. Mobile phase A was composed of 99.9 H2O (EMD Omni Solvent), and 0.1% formic acid and mobile phase B was composed of 99.9% ACN, and 0.1% formic acid. A 1 h linear gradient from 0% to 45% B was performed. The LC eluent was directly nanosprayed into an LTQ Orbitrap Velos mass spectrometer (Thermo Scientific). During the chromatographic separation, the LTQ Orbitrap Velos was operated in a data-dependent mode and under direct control of the Xcalibur software (Thermo Scientific). The MS data were acquired using the following parameters: 10 data-dependent collisional-induced-dissociation (CID) MS/MS scans per full scan. All measurements were performed at room temperature and three technical replicates of each sample were run to allow for statistical comparisons between samples, which are necessary for label-free quantification. The output was further processed using Scaffold viewer version 4.

#### Anion exchange fractionation of protein extracts

Mitotic protein extracts from nocodazole-arrested HeLa cells were loaded on a Pierce strong anion exchange spin column (Thermo Scientific). The columns were centrifuged at 500 xg for 5 minutes (at 4°C). The flow-through fraction was collected and lysis buffer was added and the columns were centrifuged again (wash step). Then, protein fractions were eluted stepwise in lysis buffer containing increasing concentrations of NaCl (50, 100, 200, 300, 400 mM). Eluted fractions were separated on SDS-PAGE gels and stained with Coomassie blue or analyzed by Western blotting (as indicated in Figure 4B). The kinase activity in the fractions was assessed in *in vitro* kinase assays, as detailed above and indicated in Figure 4C and Figure S4.

#### Knockdown of TOPK

HeLa cells were plated, cultured overnight, and then transfected with control scrambled siRNA or targeted TOPK smartpool siRNA (Dharmacon). The siRNAs were transfected into HeLa cells using DharmaFECT reagent (Dharmacon). The knockdown experiment was performed in duplicates: in one set, cells were left growing asynchronous, and in the second sets cells were synchronized with thymidine-nocodazole. Whole cell extracts were prepared, and analyzed by Western blotting, in *in vitro* kinase assays, and EMSA assays (as described above and in Figure 7).

## SUPPLEMENTARY FIGURES AND TABLES



## Abbreviations

ZFP: Zinc Finger Protein
