# Supplementary material for: Identification of the oncogenic kinase TOPK/PBK as a master mitotic regulator of C2H2 zinc finger proteins
Source: Oncotarget. 2015 Jan 13;6(3):1446–61. doi: 10.18632/oncotarget.2735 (PMC4359306; doi:10.18632/oncotarget.2735)
Supplement: Supplementary file 1 [file oncotarget-06-1446-s001.pdf]

SUPPLEMENTARY FIGURES AND TABLE

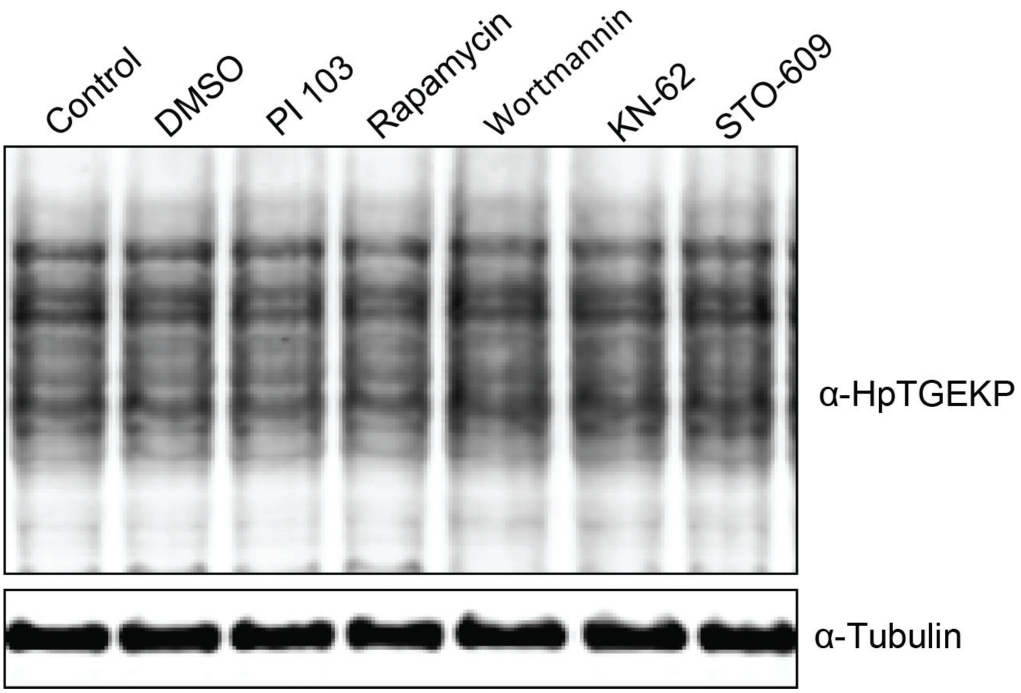

Supplementary Figure S1: Small-molecule inhibitors of the CAMK and mTOR pathways cannot inhibit mitotic linker phosphorylation. HeLa cells arrested with nocodazole for 18 hours were collected, divided into equal fractions, and treated with small molecule inhibitors, as indicated on the blot, at 1 µM concentration for 10 minutes. Cell lysates were analyzed by Western blotting and probed with anti-HpTGEKP antibody, and anti-Tubulin antibody as loading control.

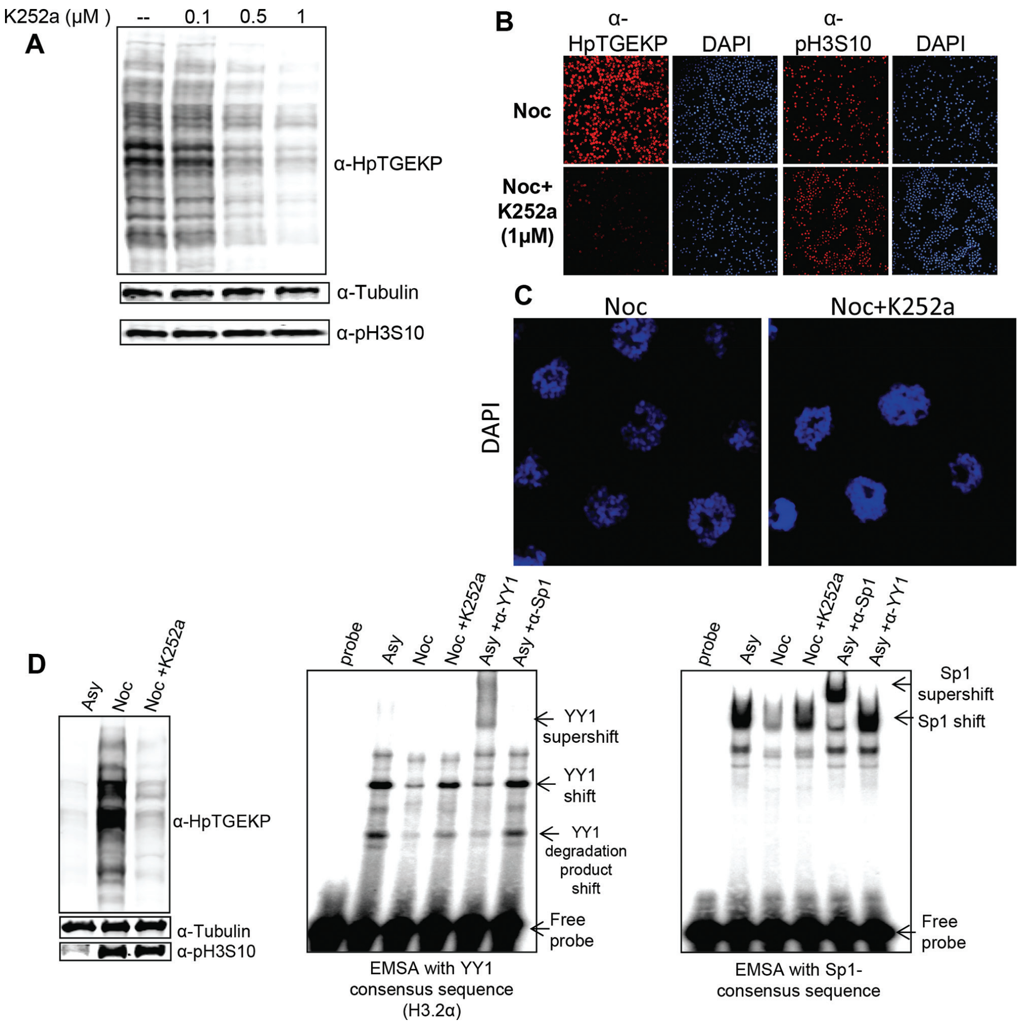

Supplementary Figure S2: K252a can inhibit linker phosphorylation without causing mitotic slippage when added at 1 µM concentration for 10 minutes. **(A)** Western blot analysis of cell lysates from nocodazole-arrested HeLa cells treated with K252a at 0.1, 0.5, and 1 µM concentrations for 10 minutes. The blot was probed with anti-HpTGEKP antibody, anti-Tubulin as loading control, and anti-pH3S10 as a mitotic marker. **(B)** Nocodazole-arrested HeLa cells were treated with K252a at 1 µM concentration for 10 minutes, and then fixed with formaldehyde and immunostained with anti-HpTGEKP or anti-pH3S10 antibodies, as indicated. Cells were stained with DAPI to visualize the nucleus/DNA. **(C)** Higher magnification of the DAPI staining of the cells in (B) shows the condensed prometaphase chromosomes. **(D)** Partial restoration of DNA binding activity of YY1 and Sp1 in protein extracts from mitotic cells treated with K252a. Cell lysates were prepared from HeLa cells growing asynchronously, or arrested with nocodazole for 18 hours, with or without treatment with K252a (1 µM for 10 minutes). The lysates were first analyzed on a Western blot with anti-HpTGEKP, anti-Tubulin (loading control), and anti-pH3S10 (mitotic marker) antibodies (left panel). The binding activity of YY1 and Sp1 proteins were assessed by EMSA assays. Cell lysates were incubated with radioactively-labeled double-stranded DNA oligonucleotides comprising the YY1 and Sp1 consensus binding sites (right panels). The YY1 and Sp1 specific shifts are indicated and confirmed by super-shift analysis with their respective specific antibodies.

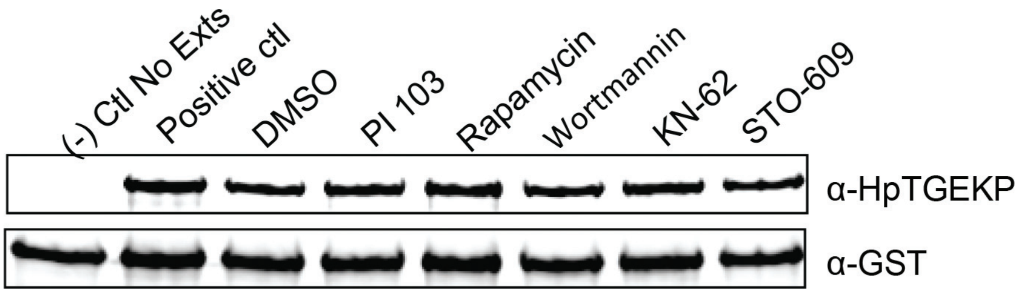

Supplementary Figure S3: Small-molecule inhibitors of the CAMK and mTOR pathways cannot inhibit linker kinase activity in mitotic extracts. Protein extracts from nocodazole-arrested HeLa cells were tested in an *in vitro* kinase assay as described in Figure 2A and 2B in the absence or presence of the indicated of small molecule inhibitors. The Western blots were analyzed by anti-HpTGEKP antibody, and anti-GST antibody to show equal substrate loading.

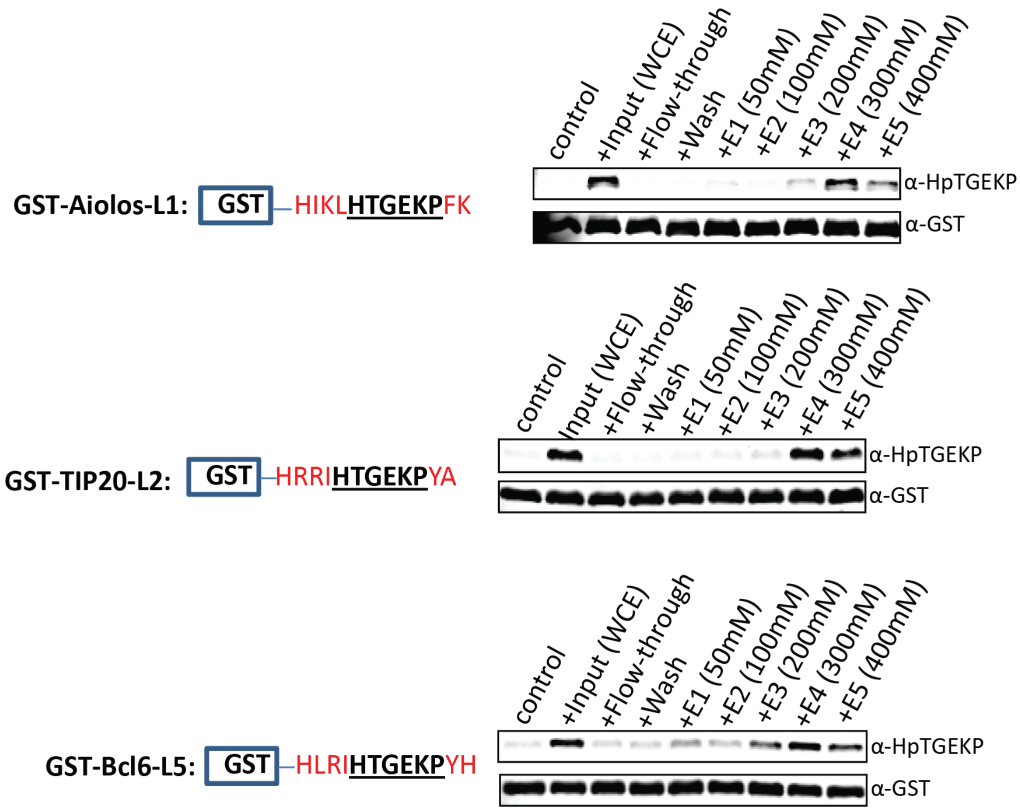

Supplementary Figure S4: Linker kinase activity of anion exchange fractionated HeLa mitotic extracts shown in Figure 4. Samples were tested in *in vitro* kinase assays with GST-tagged linker sequences of Aiolos (L1), TIP20(L2), or Bcl6(L5) coupled to glutathione beads. The kinase reactions were Western blotted and probed with anti-HpTGEK antibody ,and anti-GST antibody to show equal substrate loading.

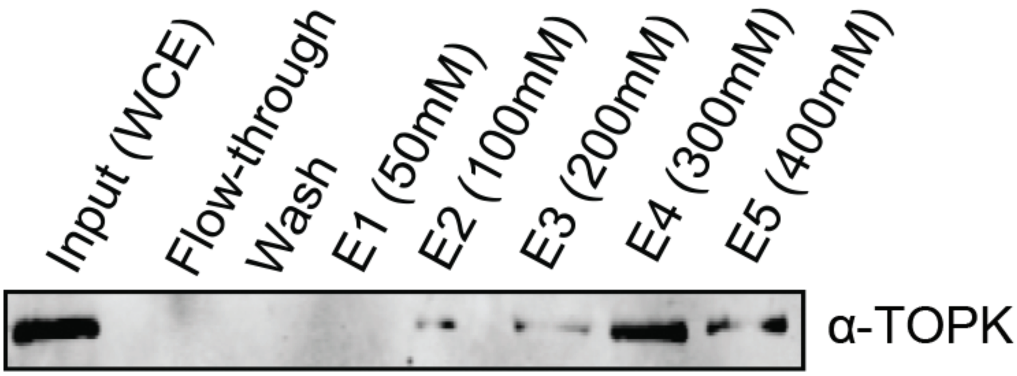

Supplementary Figure S5: Enrichment of TOPK in the anion-exchange fractions. Samples from the ion-exchange fractions (of Fig. 4B) were analyzed on a Western blot using anti-TOPK antibody. The analysis shows enrichment of TOPK in the fractions that contain linker kinase activity as tested in Fig. 4C and Fig. S4.

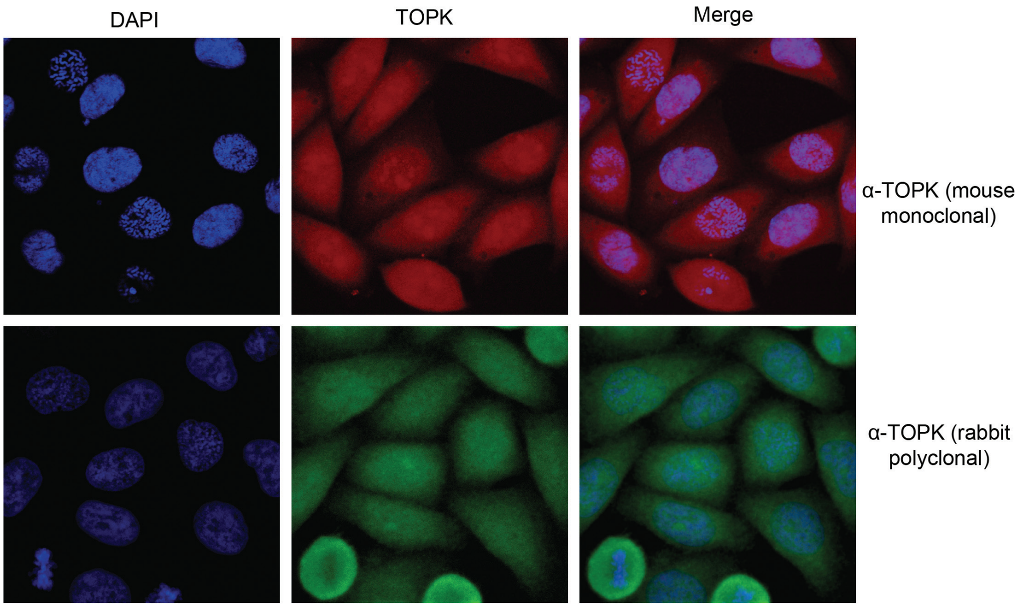

Supplementary Figure S6: Cellular localization of TOPK. HeLa cells grown on coverslips were fixed, permeabilized, and immunostained with anti-TOPK rabbit polyclonal or mouse monoclonal antibodies. Cells were stained with DAPI to visualize the nuclei.

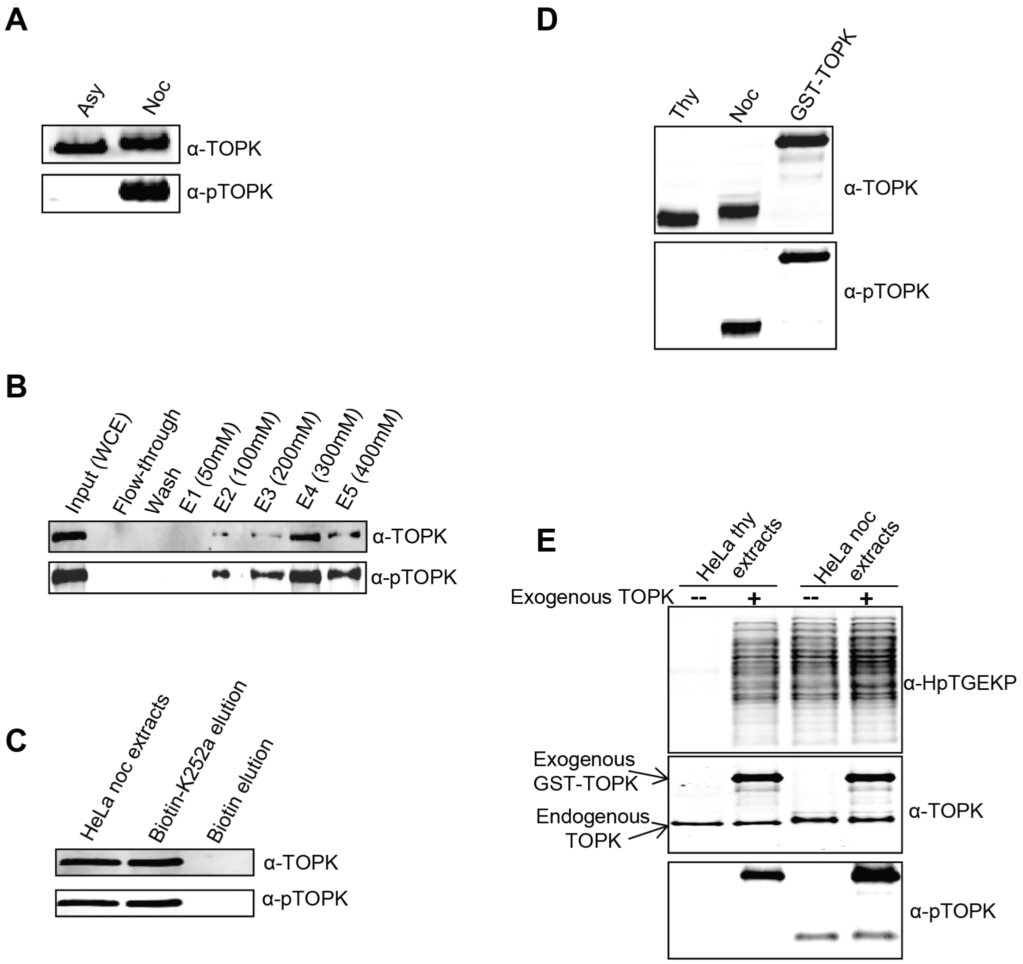

Supplementary Figure S7: Phosphorylation of mitotic TOPK and recombinant activated GST-TOPK at threonine 9. Western blot analysis with anti-TOPK and anti-pTOPK (pT9) antibodies of whole cell extracts prepared from asynchronously growing (Asy) or nocodazole-arrested (Noc) HeLa cells **(A),** anion-exchange column fraction **(B)**, and biotin-K252a elution fractions **(C)**. **(D)** Western blot analysis with anti-TOPK and anti-pTOPK (pT9) antibodies of whole cell extracts prepared from thymidine-arrested (Thy) or nocodazole-arrested (Noc) HeLa cells, and recombinant activated GST-TOPK. **(E)** Analysis of the kinase reaction of Fig. 5B with anti-pTOPK antibody showing the phosphorylation of threonine 9 on TOPK in mitotic extracts and recombinant activated GST-TOPK, but not on TOPK in thymidine extracts.

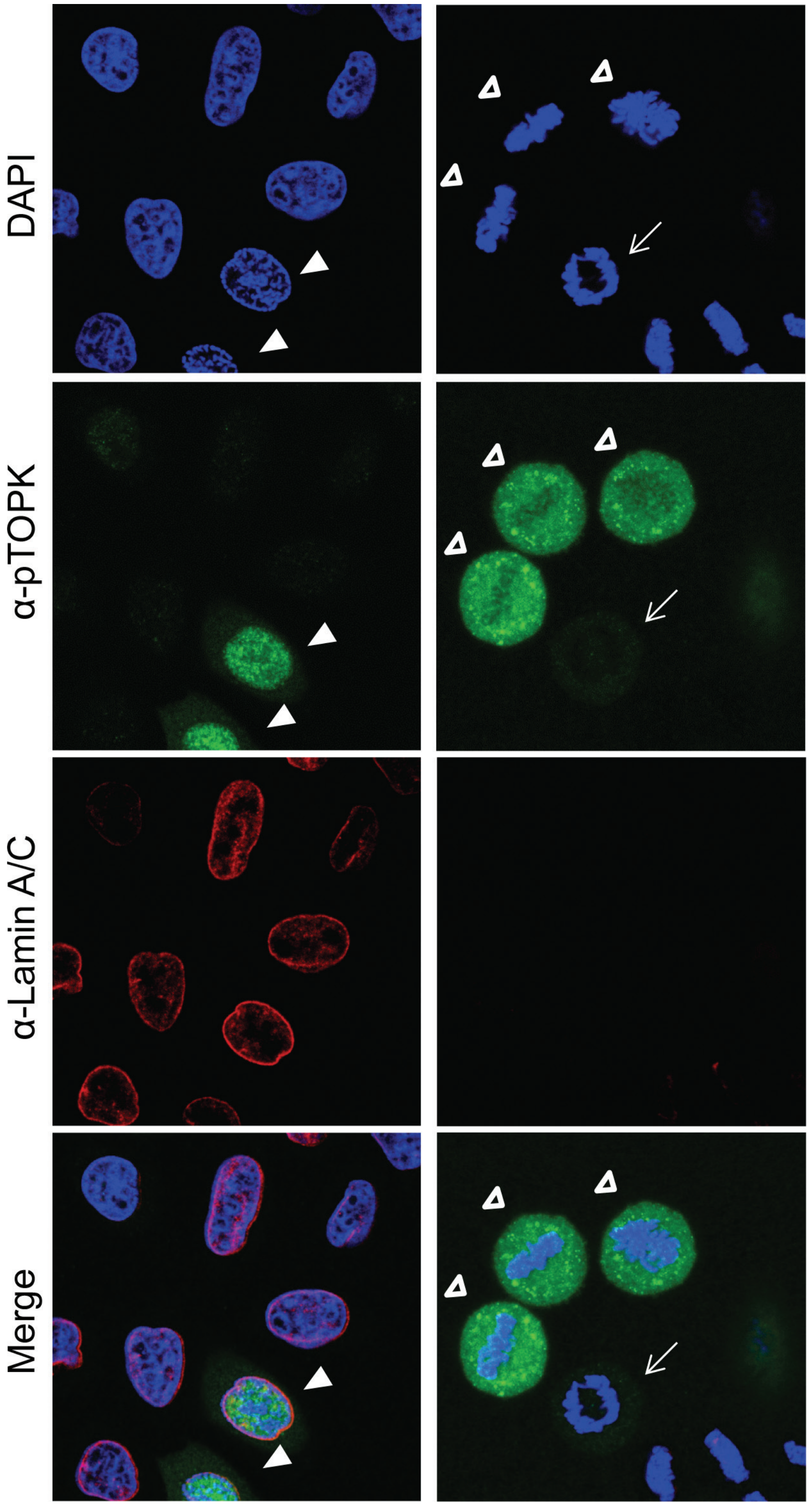

Supplementary Figure S8: Immunostaining of activated pTOPK in mitosis. HeLa cells grown on coverslips were synchronized with a single thymidine block and then released. Cells were fixed 7–9 hours after the release, permeabilized, and immunostained with anti-pTOPK antibody and with DAPI to visualize the DNA. Cells at prophase are indicated with solid arrowheads, at metaphase with empty arrowheads, and at anaphase with an arrow.

Supplementary Table S1: List of the small-molecule kinase inhibitors tested in Figure 1 and Figure S1, and their known primary target kinases according to the corresponding commercial source, as listed.

Inhibitor	Source	Primary target kinase(s)
Staurosporine (STS)	Sigma	**Pan**<br>(more than 200 kinases)
RO 31-8220	Sigma	**Broad**<br>Targets include: GRK-5, PKC(s), MAPKAP kinase 1β, S6K, AKT,CDk1, CDK2, CDK5, CDk9, CHK1, CLK, DYRK1B, EGFR, FLT3, GSK3, JAK3, KDR,MAP4K (2, 4, &5), MARK, MERTK, MINK1, Mst (1, 2, 3, &4), Trk (A, B, &C), PIM, PKN1, PRKG, PRKD, RSK, MSK, SGK, YSK1…
K252a	LC Laboratories	**Broad**<br>Targets include: PKA(s), PKC(s), PKG(s), CAMKII(s), Aurora(s), Phosphorylase kinase, Trk (A, B, &C), p42/44, Cdk1…
UCN-01	Sigma	**Broad**<br>Targets include: PKC(s), Cdk1, PAK4, Cdk5/p25 and Chk2; PDK1, lck, MAPKAP kinase-2, Akt, GSK-3β, PKA, MST2,
Roscovitine	Calbiochem	Cdk1, 2, and 5.
VX-680	LC Laboratories	Aurora kinases (can also inhibit ABL, FLT3, and KIT)
Cyclapolin 9	Sigma	Plk
RO 31-7549	Calbiochem	PKCs
SB 202190	LC Laboratories	Primary targets: p38α and β<br>Secondary targets: BRAF, Ck1, RSK4;
Bisindolylmaleimide X hydrochloride	Sigma	PKC(s)
H-89	LC Laboratories	PKA, PKCμ, PKG (also S6K, MSK)
KT5720	Sigma	PKAs
PI-103	Calbiochem	Primary targets: DNA-PK, PI3-L, mTORC1<br>Secondary targets: ATR and ATM
Wortmannin	Sigma	PI3K
Rapamycin	Sigma	mTOR
Rho-kinase inhibitor H-1152	Calbiochem	Primary target: ROCK<br>Secondary targets: CAMKII, PKG, Aurora A
TBCA	Sigma	CKII
STO-609	Sigma	CAMKK
KN62	Sigma	CAMKII
